# Charles Marchand on Peroxide of Hydrogen

**Published:** 1892-08

**Authors:** Charles Marchand


### CHARLES MARCHAND ON PEROXIDE OF HYDROGEN.

To the Editor:

Dear Sir,—I notice in the March issue of your journal an article headed "Hydrogen Peroxide," and I take the opportunity to write to you about the erroneous information contained therein.

First, in his statement, Dr. S. says that $H_2O_2$ is made by the action of peroxide of barium upon hydrochloric acid. He might have reversed and said that it was made by the action of hydrochloric acid upon peroxide of barium, which would be more correct; but this is a small matter.

The main point is, that he is wrong in his assertion, because it is not always the process which is used by a number of chemists, particularly by myself.

He further says that he has obtained from New York, Brooklyn, and St. Louis some different samples of the best makes of peroxide of hydrogen, and that the said samples were submitted to a chemical analysis by Dr. Dudley, and that Dr. Dudley found a large percentage of muriatic acid and sulphuric acid.

Referring to the preparation manufactured by me, which is the result of a special process, absolutely unknown to Dr. Dudley, as well as to any one else, he says that, "*Even this preparation contains both muriatic and sulphuric acid;*" but he leaves out the percentage question.

Now, if you kindly refer to my pamphlet, page 5, at the paragraph headed, "Important Information on Peroxide of Hydrogen," you will see that I do not attempt at all to deceive the profession about the percentage of acid which is contained in my medicinal peroxide of hydrogen; and I explain on page 6 why it contains $\frac{1}{6000}$ of muriatic acid and $\frac{1}{10000}$ of phosphoric acid, the presence of which seems not to have been noticed by Dr. Dudley. Besides that, traces of sulphuric acid are present in my medicinal peroxide of hydrogen.

In his statement, Dr. S. claims that any one of the three brands of peroxide of hydrogen submitted to this analysis had a *sufficient* quantity of muriatic acid to dissolve a *tooth* after exposure to its action.

Now, just imagine the incorrectness of his statement by figuring as follows: One pound bottle of my medicinal peroxide of hydrogen is equivalent to four hundred and fifty grammes, and the total amount of acid which is contained in one pound is two-thirds of one milligramme of muriatic acid, half a milligramme of phosphoric acid, and traces of sulphuric acid which amount to about one-tenth of a milligramme.

Now, I shall leave to Dr. S. the care of making up the calculation in order to find out the number of pounds of peroxide of hydrogen which would be required to dissolve the *smallest* tooth of an adult; and, also, the time it would require to complete the dissolution of same.

Dr. S. seems to be under the impression that muriatic acid may dissolve phosphate of lime in any proportion; unfortunately, chemistry is an exact science, and a given quanity of muriatic acid will dissolve a corresponding quantity of phosphate of lime; but nothing more than that, whatever time may be allowed for the maceration.

I will be pleased, also, if he will kindly make a calculation in order to find out the number of pounds of my medicinal peroxide of hydrogen which will represent a *sufficient* amount of free sulphuric acid to dissolve the tooth or to transform it into a biphosphate of lime which is slightly soluble, and in sulphate of lime which is practically insoluble.

When he will have done this, if he imparts to me the results of his calculation, I will make it my duty to publish it in any medical paper of which you will be kind enough to let me have the name.

I agree with Dr. S. when he says that the experiments which he has conducted have not been as *thoroughly* and as *extensively* pursued as *desired.* I am glad of it, because from the start he has been on the wrong track.

On page 205 of your journal, Dr. S. stated the evolution of gas when peroxide of hydrogen is brought into contact with pus, and he says that the same evolution of gas takes place when the pus is not present in the mouth. I regret to say that in this assertion Dr. S. is not orthodox.

Every one knows that in the mouth of all human beings a certain amount of albuminoid substances are always present, and this mere fact, that peroxide of hydrogen is brought in contact with those albuminoid substances, provokes its decomposition into water and nascent oxygen.

He says, also, that I claim that ozone is the active agent of my $H_2O_2$ medicinal. This assertion is untrue. By looking at page 3 of my pamphlet, at the article headed "Destructive Action of Ozone upon the Virus," I say that peroxide of hydrogen generates ozone, or, at least, the oxygen in a condition near to the state of ozone, as it is proven by a number of experiments of which you will find a synopsis on page 4.

Furthermore, when Dr. S. states that he has tried to obtain this evolution of gas when the $H_2O_2$ was placed in contact with pus removed from the abscess, his experiments do not show a marked success. It is possible he may have made a mistake and took some plain water, instead of peroxide of hydrogen. This is the only explanation I can give for the result reported by him.

When Dr. S. attributes the evolving of gas to the temperature of *ninety-nine degrees* which is present in the tooth, he is entirely mistaken, because when peroxide of hydrogen is heated at that temperature, the evolution of gas is hardly *perceptible.*

Following the experiment of Dr. S., when he has made an attempt to free the $H_2O_2$ from all acids, that is, muriatic and sulphuric, he evidently thought it was an easy matter to do this without decomposing the peroxide of hydrogen; but, having had some practice in the manufacture of peroxide of hydrogen, I would not undertake to perform this operation with any degree of certainty as to results.

In fact, my opinion is, that Dr. S. got rid of BOTH *acid* and *peroxide of hydrogen* which was contained in the acidulated solution. Then, of course, when he came to apply the liquid resulting from his above attempt, he could not see any evolution of gas in the canal or in the abscess. He might have repeated the same experiment a number of times, and he certainly would always meet with the same result.

Then he attributes to the muriatic acid the evolution of gas, and I wish to ask him *where the gas could originate*, since there is no gas to be generated when any acid is brought in contact with pus.

The statement presented by Professor Peirce is true. Peroxide of hydrogen acts in the way described by him. When Dr. Abbott says that he has been deluded into the idea that it was a test for the presence of pus, he seems to endorse the statement presented by Dr. S., and I am only sorry for him.

The statement of Dr. Volck, that by allowing a bottle of peroxide of hydrogen to stand open for a couple of hours, he did not find anything in it but water, shows that he had at first water, and not peroxide of hydrogen.

If you think for an instant that I have kept my medicinal peroxide of hydrogen for thirteen months in my office uncorked, without any appreciable change, you will agree with me when I say that the experiment of Dr. Volck is subject to doubt.

The statement of Dr. Fredericks is correct. That of Dr. Truman is also correct; but the assertion that peroxide of hydrogen being placed in an atmosphere above sixty degrees is rapidly decomposed and becomes water is not absolutely true. I have had several samples of my medicinal peroxide of hydrogen exposed at a temperature which varied between seventy and seventy-eight degrees for eight months. At the moment the samples were put into experiment the test showed that the preparation was $15\frac{2}{3}$ volumes capacity. At the expiration of eight months the test showed $15\frac{1}{3}$ volumes.

He is positively right when he says he does not fear the agents that are mixed with $H_2O_2$, because they are all good in their place.

There is nothing easier than to reach the bottom of the pockets of pyorrhœa alveolaris by injecting the peroxide of hydrogen by means of a glass syringe into the cavity. Whenever peroxide of hydrogen is used on cotton the results will be unsatisfactory.

Dr. S., at the end of his report, says, "I have found that where there were pus-pockets, if I could get down with a little sharpened stick, carrying the acid (muriatic) far down, it will be followed by a healthy growth and resumption of an excellent condition."

Now, I will be pleased to witness the above operation, and to follow the results which would be obtained; and I will propose to Dr. S. to submit two patients affected with the same trouble; one to his treatment, and the other to the treatment with my peroxide of hydrogen (medicinal).

I am quite certain that the results which I will obtain will be of such a satisfactory nature that he will agree with me when I say that my medicinal peroxide of hydrogen is the most powerful and, at the same time, harmless destroyer of pus which is in existence.

I think that it is my duty, as well as it is the duty of people who know something about peroxide of hydrogen, to say so, and give their opinion. This is my excuse for having taken so much of your valuable space; but I hope that you will accept my apology.

Yours, very truly,

CHARLES MARCHAND.

